# Divorce, divorce rates, and professional care seeking for mental health problems in Europe: a cross-sectional population-based study

**DOI:** 10.1186/1471-2458-10-224

**Published:** 2010-04-29

**Authors:** Piet F Bracke, Elien Colman, Sara AA Symoens, Lore Van Praag

**Affiliations:** 1 Department of Sociology, Ghent University, Belgium, Korte Meer 5, 9000 Ghent, Belgium

## Abstract

**Background:**

Little is known about differences in professional care seeking based on marital status. The few existing studies show more professional care seeking among the divorced or separated compared to the married or cohabiting. The aim of this study is to determine whether, in a sample of the European general population, the divorced or separated seek more professional mental health care than the married or cohabiting, regardless of self-reported mental health problems. Furthermore, we examine whether two country-level features--the supply of mental health professionals and the country-level divorce rates--contribute to marital status differences in professional care-seeking behavior.

**Methods:**

We use data from the Eurobarometer 248 on mental well-being that was collected via telephone interviews. The unweighted sample includes 27,146 respondents (11,728 men and 15,418 women). Poisson hierarchical regression models were estimated to examine whether the divorced or separated have higher professional health care use for emotional or psychological problems, after controlling for mental and somatic health, sociodemographic characteristics, support from family and friends, and degree of urbanization. We also considered country-level divorce rates and indicators of the supply of mental health professionals, and applied design and population weights.

**Results:**

We find that professional care seeking is strongly need based. Moreover, the divorced or separated consult health professionals for mental health problems more often than people who are married or who cohabit do. In addition, we find that the gap between the divorced or separated and the married or cohabiting is highest in countries with low divorce rates.

**Conclusions:**

The higher rates of professional care seeking for mental health problems among the divorced or separated only partially correlates with their more severe mental health problems. In countries where marital dissolution is more common, the marital status gap in professional care seeking is narrower, partially because professional care seeking is more common among the married or cohabiting.

## Background

The increase in the divorce rate has received a great deal of scientific attention. Most research has focused on the consequences of divorce or separation for ex-partners and their children. The health consequences of divorce are also well documented, showing the detrimental effects of divorce on both somatic [1-5] and mental health [2,6-9], with the divorced or separated experiencing higher levels of depression, stress, and fear, as well as lower levels of self-esteem [10-20]. However, little is known about how these adverse mental health consequences translate into the use of health care services. A few existing studies have indicated that mental health care use and health care use in general is higher among the divorced or separated than among the married or cohabiting, regardless of mental health status [21-24]. Compared to the married or cohabiting, the divorced or separated visit professional health care providers like general practitioners [21], specialists [21], and psychiatrists [22] more often, and are also hospitalized more often [21,25]. Single mothers seem to suffer the most; they have more health problems and turn to professional mental health services more frequently [26-29]. Moreover, Dutch research has indicated that the divorced or separated not only have higher rates of mental health care use, they also have higher rates of unmet need. This means that they are more often in a situation in which they have mental health care needs but do not receive sufficient care [30]. On the other hand, for a subgroup of single mothers, a Canadian study found that the higher use of professionals for mental health reasons reflected their higher rates of psychopathology [29]. Apart from Bijl and Ravelli's study [30], the aforementioned studies were not based on a representative sample of the general population, did not adjust for important intermediary variables or confounders like socioeconomic status and the availability of informal support [25], or did not include data on anyone other than women [25]. Furthermore, existing studies have focused only on a single country--the United States of America [22], the Netherlands [30], Canada [29,31], or the United Kingdom [25]. Moreover, only two studies [25,30] have focused on care seeking for mental health problems. In this study, we examine whether there are differences between the divorced and the married in professional care seeking due to mental health problems, using a representative sample of the population of 29 European countries. Based on Andersen's Behavioral Model [32], we are able to identify some social structural determinants that explain these individual differences in mental health care consumption at both the individual and the country level. Andersen's Behavioral Model asserts that the consumption of medical care depends on the presence of predisposing characteristics, enabling resources, and need factors.

*Predisposing characteristics *are those features that are present before the development of a mental health problem. We consider household composition, education, work status, age, and gender to be relevant predisposing features.

The predisposing characteristic that is our main interest is household composition--more specifically, marital status. A few studies that exist show that different family compositions are associated with different amounts of health care use [25,33] and demonstrate that there is a higher mental health care consumption among the divorced or separated than the married or cohabiting [21-23,34]. Another relevant aspect of household composition is the presence of children. Because divorced parents are forced to maintain contact with each other, the presence of children in the household may have an impact on their mental health. In addition, having sole custody of children often involves parenting strain and financial costs, making it more difficult to find a job, make new friends, or find a new partner [35-37], all of which act as important buffers against mental health problems (e.g., [2]). On the other hand, children may add a sense of meaning to parents' lives, and older children in particular may be an important source of practical and social support for their parents [38,39].

Educational level also affects the amount of mental health care consumption sought. Even after controlling for mental health status, the lower educated are less inclined to turn to professional mental health care services [24]. Previous research has indicated that divorce is more common among the highly educated [40], which may explain the higher mental health care use among the divorced. Recently, however, some authors [41,42] have argued that in countries where the cost of divorce has decreased, the lower educated are more at risk of divorce, undermining that explanation.

Work status also correlates with the use of mental health care: compared to working people, people outside of the labor market, like the unemployed, the retired, and housewives and househusbands, are found to be less inclined to seek professional care because of mental health problems [24,43], even after mental health status is controlled for. Consequently, since divorced people have an increased risk of being laid off [44,45], we could expect them to seek professional mental health care less often.

With regard to age, the risk of illness increases with age and, consequently, health care consumption expands [43,46].

Research has also shown that women have higher health care utilization rates than men [24,46,47]. Some research indicates that this is largely explained by the existence of gender differences in mental health regardless of marital status [46]. Some researchers assert that being divorced or separated has a more severe impact on the mental health of women than of men [12,48], which may explain higher mental health care consumption by divorced women. Others explain these gender differences in service use by pointing out that women are generally found to be more inclined to seek professional help, even after their actual need is controlled for [49]. According to these researchers, the impact of marital status on mental health care consumption might therefore differ by gender, with women having a higher mental health care use rate than men.

*Enabling resources *are the means and knowledge needed to acquire care. To receive care, one has to possess both financial means and the knowledge of where to go to seek help. In Europe, the wealthy are more likely to seek out specialist care, while access to primary care seems to be pro-poor or equal for all income groups, even after controlling for need [50,51]. In addition, social support from relatives and friends can be considered an enabling resource. On the one hand, social support can encourage health care use, as social networks may help with the recognition of the development of health problems and may stimulate a person to seek professional care [32,52-54]. On the other hand, social support may also impede health care consumption. Individuals experiencing problems usually turn to their immediate environment for help before contacting formal care services. However, as individuals with low levels of social support cannot rely on the help of their acquaintances, asking for professional care can be a way to compensate for a lack of social support [32,55]. Compared to the married and cohabiting, the divorced have smaller social networks and less social support available to them [56]. In addition to the loss of their former partner, they also lose half of their relatives and, often, some shared friends [38]. We therefore expect the divorced to have higher mental health care use due to less available social support.

*Need factors *include health status and the perceived need for help [21,32]. On average, the divorced, compared to the married, have worse somatic [2,4,5,21] and mental health statuses. They experience higher levels of distress, depression, and anxiety [2,6,7], and have to deal with certain somatic problems more frequently [2], often putting an extra burden on their mental health. The differences in mental and physical health can be largely--but not completely--explained by a socioeconomic situation that is worse and by a lack of social support [2,3,57]. Other explanations are health selection effects--healthier individuals are less likely to divorce [2,4,58], the damaging effects of the stigmatization of divorce, and the occurrence of negative life events as a consequence of divorce, for example, having to move [2]. Thus, the higher health care consumption of the divorced may be in large part a consequence of worse physical and/or mental health. However, even after controlling for these need factors, previous research indicates that the mental health care consumption rate of the divorced seems to remain higher than that of the married [21,25]. Moreover, persons without a partner express higher unmet care needs than those living with a partner [30].

To control for between-country differences in the distribution of our key variables--*marital status *and *professional care consumption*--we consider two country-level indicators, the country-level *divorce rates *and *the supply of professional care*.

We expect to see differences between countries in the use of mental health services on the basis of marital status, because norms and values about family and divorce as well as social policies about marital dissolution and sole parenthood vary between countries [59]. At present, there is insufficient research on the link between the divorce rate and marital status differences as they relate to mental health service use. However, we see two possible ways in which the divorce rate might interact with the relation between marital status and mental health services use. First, we expect to find that higher divorce rates go hand in hand with an enhanced professional service use by the divorced. Although more subtle forms of stigmatization are still possible [60], one can expect divorce to be less stigmatized in countries where the divorce rate is higher [61], making it easier for the divorced to seek help [62]. Second, there are indications that the use of professional care among the divorced could actually be lower in countries with a high divorce rate. On the basis of social comparison theory [62] and of research on divorce and stigma [60], we can assume that people in high-divorce countries will no longer view marital dissolution as different or problematic [7,63-65], resulting in a relative decline in professional care seeking among the divorced. Of course, it is also possible that the divorce rate has no effect on professional care consumption beyond the compositional effects that result from the marital status composition of the population.

The *availability of mental health services *may also influence utilization [66]. We expect that the use of professional mental health care will be greater in countries where help is available and accessible. According to Wang et al. [67], unmet need is worse in low- and middle-income countries, and the treatment gap may therefore be attributable to the reduced amount these nations have to spend on mental health care from health budgets that are already overburdened [66]. Consequently, we can expect cross-national variation in the availability of mental health services to influence utilization, even among middle- and high-income European countries. Hence, the availability of general or more specialized professionals is a supply-side factor that should not be ignored [68] and that could be incorporated into Andersen's model as an important enabling factor at the supra-individual level. Country-level indicators are unable to accommodate important within-country differences in the availability of mental health services. In order to consider these within-country differences, we also include the degree of urbanization in the area of residence as a rough indicator of the availability of more specialized mental health professionals [68]. Differences between rural and urban areas may also signal differences in stigma beliefs. For instance, as Hoyt and colleagues [50] have shown, in the rural areas of the United States a reluctance to seek professional help is closely linked to stigmatizing beliefs about mental health care.

In sum, we will first examine whether a difference exists between the married or cohabiting and the divorced or separated in their use of professional health care for emotional or psychological problems. Second, we will try to uncover some predisposing, enabling, and need factors that explain this possible difference in amount of use. Finally, we intend to examine between-country variation by introducing country characteristics--divorce rate and the supply of services--into the analysis.

## Method

### Sample

We derived data from multistage national probability samples of the general population of 29 European countries collected during the Eurobarometer 248 survey between December 2005 and January 2006. The sampling design in all participating countries was based on a random selection of sampling areas stratified by urbanization (the distribution of metropolitan, urban, and rural areas). A cluster of addresses were selected from these sampled areas. In each household at a sampled address, a respondent was selected using a random selection procedure [69]. Telephone interviews were conducted with European Union citizens residing in the 25 member countries, with the citizens of the two then-acceding countries Bulgaria and Romania, and with the two candidate countries Croatia and Turkey including the citizens of the Turkish Cypriot Community. In our analyses, we merged the data from East and West Germany, from Northern Ireland and the United Kingdom, and from the Republic of Cyprus and the Turkish Cypriot Community. For the present analysis, we limited the sample to respondents aged 21 and older; respondents from all marital status categories were included. We applied population weights so that each country was represented in proportion to its population size, and we considered country-specific design weights to correct for differences within national populations in gender, age, region, and size of locality.

The resulting samples are representative of the population of citizens in the included countries aged 21 and over. In total, 27,146 respondents were included (11,728 men and 15,418 women). Table 1 presents descriptive statistics of the weighted sample.

**Table 1:** Individual-level descriptive statistics.

	Total		Men		Women	
	**N**	**%**	**N**	**%**	**N**	**%**

**Marital status**						
Married/Cohabitants (ref.)	17549	64.6	8179	69.7	9370	60.8
Divorced/Separated	3210	11.8	1233	10.5	1977	12.8
Single	2832	10.4	1556	13.3	1276	8.3
Widowed	3318	12.2	647	5.5	2671	17.3
Other	237	0.9	113	1.0	124	0.8
**Education in years**						
≤15 years or other	6897	25.4	2720	23.2	4177	27.1
16--19 years	11573	42.6	5081	43.3	6492	42.1
≥20 years (ref.)	6928	25.5	3221	27.5	3707	24.0
**Work status**						
Manual workers (ref.)	5656	20.8	2947	25.1	2709	17.6
Self-employed	2136	7.9	1385	11.8	751	4.9
Managers/professionals	2771	10.2	1432	12.2	1339	8.7
White-collar workers	2898	10.7	1194	10.2	1704	11.1
Housewives/Househusbands	3006	11.1	92	0.8	2914	18.9
Unemployed	1654	6.1	689	5.9	965	6.3
Retired	8147	30.0	3592	30.6	4555	29.5
Still studying	878	3.2	397	3.4	481	3.1
**First health support**						
from family	13507	49.8	5699	48.6	7808	50.6
from friends	5445	20.1	2083	17.8	3362	21.8
**Urbanization**						
Rural area or village	10400	38.3	4439	37.8	5961	36.4
Small- or middle-sized town	9567	35.2	4161	35.5	5406	35.1
Large town	7363	27.1	3078	26.2	4285	27.8

**(range)**	**Mean**	**SD**	**Mean**	**SD**	**Mean**	**SD**

**Professional support **(0--7)	0.18	0.50	0.15	0.467	0.21	0.53
**Mental well-being **(1--5)	3.63	0.74	3.75	0.70	4.93	0.87
**Physical health **(1--5)	3.97	1.14	4.11	1.09	3.87	1.17
**N of children **(0--15)	0.46	0.84	0.41	0.80	0.49	0.87
**N of other adults **(15+) (1--6)	0.62	0.99	0.62	1.00	0.62	0.98
**Age **(21--98)	49.6	17.1	49.1	16.9	49.9	17.2

### Variables

#### Professional care use

The dependent variable *professional care use *was measured by the total number of different health professionals--general practitioner, psychiatrist, psychologist, psychoanalyst, nurse, social worker, or psychotherapist (not mentioned previously)--that respondents had contacted for an emotional or psychological health problem during the last 12 months. The total number of these events followed a Poisson distribution. Most people (85.9%) had not contacted a health professional because of emotional or psychological problems during the past 12 months.

#### Health status

We included both physical and mental health status in the analysis. *Physical health *was measured by compiling the answers to the question "During the past 4 weeks how much of the time have you had any of the following problems with your work or other regular activities as a result of your physical health?" Respondents could address the two items--"you have accomplished less than you would like" and "you have accomplished your usual activities less carefully"--using responses ranging from *all the time *through *never*. A higher score indicates better physical health. Our physical health indicator consists of the mean score of these two items. Scores on both items correlate very strongly with Pearson's r = 0.85 (p < 0.001). The bivariate correlation exceeds 0.76 in all participating countries except Denmark, where a correlation coefficient of 0.66 still points to a strong association between both items. *Mental health *was based on the 36-Item Short-Form Health Survey [70], which includes both a mental health (5 items, the MHI-5) and an energy/vitality (4 items, the EVI scale) dimension. The scale used here consists of the mean of all nine item scores, and gives an indication of how the respondents have felt and how things went during the last 4 weeks, with a higher score indicating a better mental health status. A Cronbach's alpha of 0.89 demonstrates its reliability; Cronbach's alpha exceeds 0.84 in all participating countries.

Information on physical health was lacking for 184 respondents (0.7% of the sample); while 108 respondents (0.4% of the sample) failed to provide answers to at least six items of the mental health dimension. Full information on these two health status measures was available for 99.3% and 97.3%, respectively, of the sample. For both health status indicators, the overall item mean scores replaced missing values.

#### Predisposing characteristics

*Marital status *is the main independent variable in this study. In Table 1, we compared respondents who are divorced or separated (11.8%), single (10.4%), or widowed (12.2%), as well as a generic category of unidentified others (0.9%) to those who are married or who cohabit (64.6%). The category of unidentified others contains respondents who spontaneously classified themselves in this rest category. It is important to note that we measured the *current *marital status: respondents who were divorced or separated but were living together with a new partner were categorized as married or cohabiting.

The *number of children *(up to 15 years old), and the *number of adult household members *(15+) in addition to the spouse/partner were added. The reference group was a household that had no other members besides the spouse/partner. The data did not allow us to distinguish between children aged 16 and over and others of comparable age living in the household.

The number of years of *education *the respondents had completed was subdivided into the following categories (see Table 1): 20 years or more (25.5%), 16 through 19 years (42.6%), and less than 16 years (25.4%). Those who had studied 20 years or more were set as the reference category. Separate categories were added for those still studying (3.2%) and those whose educational level is unknown (3.3%).

*Work status *was measured on the basis of social class and employment status, using eight categories (see Table 1): managers/professionals (10.2%), white-collar workers (10.7%), the self-employed (7.9%), manual workers (20.8%), the unemployed (6.1%), the retired (30.0%), students (3.2%), and housewives and househusbands (11.1%). Manual workers were set as the reference category.

*Age *was measured in years and had a mean of 49.6 years with a standard deviation of 17.1 years (see Table 1).

#### Enabling resources

Our measure of social support was based on the question "Who would you contact for *first health support*?" Multiple answers were possible. Two dichotomous indicators were constructed (see Table 1) indicating respondents who mentioned they would seek support *from their family *(49.8%), and, in a separate variable, *from their friends *(20.1%).

The measure of the degree of *urbanization *of the place of residence was based on the question "Would you say you live in a rural area or village, a small- or middle-sized town, or a large town?" People living in a rural area or a village were set as the reference category.

#### Country-level characteristics

Using information provided by Eurostat [71], we determined country-level divorce rates for 2005. Like other research, the current study used area provider density scores to measure supply-side effects [72,73].

We constructed an overall indicator of the supply of professional care by totaling the number of general practitioners per 100,000 inhabitants, the number of psychiatrists and psychologists per 100,000 inhabitants, and the number of psychiatric nurses or social workers in mental health settings per 100,000 inhabitants [74].

### Analysis

Hierarchical linear models were estimated using HLM version 6.0 [75]. We based our outcome variable on the number of occurrences of a relatively rare event, namely, consulting a health professional. Therefore, due to the nature of the dependent variable, Poisson regression analyses were appropriate [75]. We added an estimate of overdispersion, σ^2^, to the equations to adjust for the dependence of the variance on the mean of the outcome indicator [76]. The intra-class correlation is 0.064 (=0.096/[0.096 + 1.41]), therefore 6.4% of the total variance in the outcome is between-countries variance (Chi^2 ^[df = 28] = 309.2, p < 0.001; no Table).

All variables were grand mean centered. As mentioned above, both population and design weights were applied.

First, a baseline model was estimated, showing the results of marital status and gender as well as the interaction effect with gender and being divorced or separated (Model 1). We added random slope estimates to account for between-country variation in the effects of divorce on the outcome variable professional mental health care for both women and men.

After need factors (Model 2), predisposing characteristics and enabling resources (Model 3) were introduced. In the final model (Model 4), country characteristics and the various cross-level interactions between divorce rate and the different marital status categories were added. Also, all cross-level interactions between our supply of professional care indicator and the different marital status categories were added in addition to the random slope estimates of all marital status categories. Estimations of this more elaborate model show significant between-country variations between all marital status categories and professional care use, but show no significant cross-level interaction effects with the supply of professional care. Therefore, a final, more parsimonious, model is estimated (Model 5) after elimination of the latter cross-level interaction effects.

The country-level descriptive statistics are shown in Table 2. Information on the supply of psychiatric nurses or social workers in mental health settings was lacking in two countries, Belgium and Croatia. Therefore, the country-level supply of professional care indicator for these countries was constructed after substituting overall mean scores for missing values. We controlled for the possible effects of this missing values substitution by adding a dichotomous indicator that identifies both countries with partially missing information on the supply of professionals. The results of this analysis were very close to the results reported in Tables 3 and 4: All coefficients showed similar levels of significance and the aforementioned indicator of missing value substitution exerted no significant effect (γ = 0.054, SE = 0.263, p = 0.838).

**Table 2:** Country-level descriptive statistics.

Country	N	Divorce Rate	Care supply	
Austria	971	2.4	572	
Belgium	914	2.9	560.5	(*)
Bulgaria	922	1.9	55.2	
Croatia	935	1.1	381.2	(*)
Cyprus	923	2	324.3	
Czech Republic	936	3.1	467.6	
Denmark	988	2.8	527	
Estonia	910	3	419.6	
Finland	952	2.6	761	
France	977	2.5	522.6	
Germany	1442	2.4	932.3	
Greece	948	1.2	588	
Hungary	963	2.5	331	
Ireland	922	0.8	493.2	
Italy	928	0.8	422.3	
Latvia	931	2.8	362.5	
Lituania	916	3.3	513.6	
Luxemburg	476	2.3	380	
Malta	455	0	501.7	
Poland	911	1.8	228.4	
Portugal	932	2.2	358.4	
Romania	934	1.5	265.1	
Slovakia	956	2.1	356	
Slovenia	940	1.3	253.25	
Spain	922	1.7	397.3	
Sweden	945	2.2	515.6	
The Netherlands	1069	2	682	
Turkey	897	1.3	166	
UK and Northern Ireland	1231	2.6	412	

**Table 3:** Determinants of professional care seeking (HLM Poisson regression): individual effects

	Model 1: Baseline			Model 2: Need indicators added			Model 3: Full individual level model			Model 4: Country level and cross-level indicators added			Model 5: More parsimonious model (Supply indicator removed)		
	Coeff.	SE	P	Coeff.	SE	P	Coeff.	SE	P	Coeff.	SE	P	Coeff.	SE	P
**Intercept**	-1.781	0.055	***	-1.998	0.077	***	-2.006	0.075	***	-2.017	0.067	***	-2.016	0.067	***

**INDIVIDUAL-LEVEL VARIABLES**															
**PREDISPOSING CHARACTERISTICS**															
**Marital status (Married/Cohabitants = ref.)**															
Divorced/Separated	0.588	0.115	***	0.366	0.092	***	0.339	0.096	**	0.378	0.099	***	0.377	0.098	***
Single	0.024	0.094		0.090	0.070		0.070	0.079		0.080	0.087		0.080	0.086	
Widowed	0.456	0.066	***	-0.093	0.065		-0.072	0.067		-0.042	0.078		-0.043	0.077	
Other	0.183	0.268		0.048	0.222		-0.070	0.237		-0.096	0.240		-0.031	0.228	
**Gender (Men = ref.)**															
Women	0.303	0.055	***	0.108	0.053		0.096	0.053		0.094	0.050	*	0.093	0.050	*
Women*divorced/separated	-0,039	0.126		-0.020	0.100		-0.014	0.103		-0.014	0.108		-0.013	0.108	
**Number of children up to 15 years**															
**Number of other adults**							-0.026	0.018		-0.210	0.018		-0.210	0.018	
**Education (≥20 years = ref.)**															
16--19 years							0.001	0.060		-0.012	0.042		-0.012	0.042	
≤15 years or other							-0.037	0.062		-0.050	0.049		-0.050	0.049	
Still studying							-0.206	0.338		-0.209	0.115	*	-0.210	0.115	*
Don't know							0.027	0.118		0.009	0.102		0.011	0.102	
**Work status (Manual workers = ref.)**															
Self-employed							0.140	0.096		0.115	0.071		0.116	0.071	
Managers/professionals							0.036	0.104		0.033	0.069		0.035	0.069	
White-collar workers							-0.123	0.114		-0.119	0.068	*	-0.119	0.068	*
Housewives/househusbands							0.140	0.083		0.136	0.066	**			
Unemployed							0.165	0.093		0.170	0.067	**	0.171	0.067	**
Retired							0.246	0.082	**	0.247	0.058	***	0.247	0.058	***
**Age**							-0.005	0.003	*	-0.005	0.001	***	-0.005	0.001	***
**ENABLING RESOURCES**															
**First health support**															
from family							0.043	0.043		0.042	0.032		0.043	0.032	
from friends							0.079	0.059		0.079	0.038	**	0.078	0.038	**
**Urbanization**							0.050	0.034		0.047	0.020	**	0.048	0.020	**
**NEED FACTORS**															
**Mental well-being**				-0.802	0.055	***	-0.795	0.055	***	-0.794	0.023	***	-0.795	0.023	***
**Physical health**				-0.195	0.024	***	-0.192	0.024	***	-0.192	0.016	***	-0.192	0.016	***

**Table 4:** Determinants of professional care seeking (HLM Poisson regression): country level effects and variance components

	Model 1:			Model 2:			Model 3:			Model 4:			Model 5:		
	Coeff.	SE	P	Coeff.	SE	P	Coeff.	SE	P	Coeff.	SE	P	Coeff.	SE	P
**Divorce rate**										0.094	0.087		0.093	0.086	
Divorce rate*divorce/separated										-0.188	0.084	**	-0.188	0.080	**
Divorce rate*single										0.127	0.111		0.112	0.107	
Divorce rate*widowed										0.026	0.100		0.029	0.095	
Divorce rate*other										0.183	0.367		0.063	0.329	
**Supply of health professionals**										0.001	3.39^E^-4		0.001	3.38^E^-4	
Supply of health professionals*divorce/separated										-4.5^E^-5	3.42^E^-4				
Supply of health professionals*single										1.90^E^-4	4.95^E^-4				
Supply of health professionals*widowed										6.2^E^-5	4.22^E^-4				
Supply of health professionals*other										-0.002	0.002				
**Variance Components**	**VC**	**Chi**^**2**^	**P**	**VC**	**Chi**^**2**^	**P**	**VC**	**Chi**^**2**^	**P**	**VC**	**Chi**^**2**^	**P**	**VC**	**Chi**^**2**^	**P**

**Intercept (df = 28)**	0.084	264.4	***	0.142	443.4	***	0.142	448.4	***	0.120	342.1	***	0.119	340.7	***
**Marital status**															
Divorced/Separated	0.207	71.5	***	0.126	62.8	***	0.120	60.9	***	0.148	61.2	***	0.144	61.3	***
Single										0.115	58.7	***	0.110	58.5	***
Widowed										0.101	55.8	***	0.095	55.6	***
Other										0.654	51.9	***	0.640	54.3	***
**Gender (df = 28)**	0.050	58.1	***	0.038	54.2	**	0.038	54.4	**	0.027	43.3	**	0.027	43.3	**
Divorced/Separated*Gender	0.252	59.2	***	0.143	52.0	**	0.142	51.1	**	0.138	45.8	**	0.138	45.7	***
**Sigma**	1.381			1.178			1.171			1.156			1.075		

Because of the large sample size, we set the minimum level of significance at p < 0.001 for the individual-level effects, and at p < 0.05 for the country-level and the cross-level effects.

## Results

The estimation of the baseline model shows that, compared to the married or cohabiting, the divorced or separated (Table 3, Model 1: B = 0.59, SE = 0.114, p < 0.001; event rate = 1.80 [95% CI 1.42, 2.27]) and the widowed (Table 3, Model 1: B = 0.456, SE = 0.066, p < 0.001; event rate = 1.58 [95% CI 1.39, 1.80]) visit professional health care providers more often when facing emotional or psychological health. Furthermore, women consult more health care professionals than men do (Table 3, Model 1: B = 0.303, SE = 0.055, p < 0.001; event rate = 1.35 [95% CI 1.21, 1.51]). Despite these significant effects, no overall divorce*gender interaction effect was found concerning the use of mental health care (Table 3, Model 1: B_gender*divorced _= -0.039, SE = 0.126, p = 0.756). Significant random slope coefficients are present for all core variables (Table 3, Model 1: μ_divorce _= 0.21, p < .001, SD = 0.46; μ_gender _= 0.05, p < .01, SD = 0.22; μ_divorce*gender _= 0.25, p < .01, SD = 0.50), showing significant between-countries variation in the aforementioned findings: In some countries the aforementioned findings do not hold, while in other countries they are more pronounced. For instance, our baseline model estimates that in approximately 20 countries the effect of divorce or separation on professional care use falls within a range of 1.05 > B_divorce _> 0.13.

After including both somatic and mental health status (Table 3: Model 2), some of the aforementioned marital status and gender differences in professional care use are found to be attenuated. Gender differences in professional care seeking seem to be largely need based (B = 0.108, SE = 0.053, p = n.s.). The more frequent professional care consumption of the widowed also coincides with their self-reported health status (B = -0.093, SE = 0.065, p = n.s.). In contrast, the professional care seeking of the divorced or separated is only partially accounted for by their somatic and mental health status (B = 0.366, SE = 0.092, p < 0.001).

These findings remain consistent after introducing the predisposing factors (sociodemographic controls) and enabling resources (the availability of support from friends and family) into the model (Table 3: Model 3). Compared to the employed, both the unemployed (B = 0.165, SE = 0.093, p < 0.05) and the retired (B = 0.246, SE = 0.082, p < 0.01) seek professional support for mental health problems more often. Younger adults also rely on professionals more often (B = -0.005, SE = 0.003, p < 0.01). Adding these individual-level indicators to the baseline model does not substantially alter the strength of the random coefficients.

Finally, in Model 4 we added the two country-level indicators and the accompanying cross-level interaction effects with divorce and the other marital status categories (see Table 3 and 4). The country-level supply of health professionals does not exert any substantial effect on the use of professional care. In contrast, differences in professional care use between the divorced or separated and the married or cohabiting depend heavily on the country-level divorce rates: In countries with high rates of marital dissolution, the marital status differences largely disappear (B_divorce _= 0.378, SE = 0.099, p < 0.001; γ_divorcerate*divorce _= -0.188, SE = 0.084, p < 0.01). The estimates from the more parsimonious model (Model 5) confirm these results.

Based on Model 5, we estimated the predicted number of visits to health professionals for the married or cohabiting and the divorced or separated at 0.11 and 0.23, respectively, in countries with low overall divorce rates (mean minus 2 std. dev.), at 0.13 and 0.19, respectively, in countries with mean overall divorce rates, and at 0.16 and 0.16, respectively, in countries with high overall divorce rates (mean plus 2 std. dev.). These estimated scores make it clear that in countries with a high divorce rate the marital status gap in professional care use is smaller because of not only lower care use among the divorced or separated, but also because of higher care use among the currently married and cohabiting.

## Discussion

Using data from the Eurobarometer 248, we estimated marital status differences in professional care use for mental health problems in a sample of 27,146 inhabitants of 29 European countries, 21 years and older. We found that the divorced or separated, compared to the married or cohabiting, seek professional support more often irrespective of mental and physical health status, and irrespective of social background (education, work status) or of available informal support. Moreover, these marital status differences vary from country to country. We found large differences in countries with low overall divorce rates, irrespective of the availability of health professionals. In countries with higher divorce rates, the marital status gap largely disappears not only because professional care seeking among the divorced is lower, but also because the married or cohabiting seek professional support more frequently. Before interpreting our results, some limitations should be considered.

First, financial means are an important enabling resource in regard to professional care [32]. Overall, low-income groups were found to use fewer mental health services, after controlling for mental health status [19,49,59], although important differences appear when comparing general and specialized professional support. In the present study, we were unable to consider income differences due to the limitations of the dataset used. This meant that an important aspect of the explanation for higher mental health care use for the divorced or separated was missing, given that the divorced or separated--especially single parents--experience a more stringent financial situation [60-62]. Nevertheless, the indicators of education and work status may at least partially replace these income effects. Moreover, because we expect mental health professional care seeking to be less among low-income groups, the inability to consider the financial situation statistically could actually lead to an underestimation of the marital status differences in professional care seeking.

Second, our dependent variable reflected both general and specialized professional care use for emotional or psychological health problems. By using such a broad indicator, we were not able to explore marital status differences in general vis-ΰ-vis specialized professional care. The low prevalence of specialized professional care use in most marital status categories in the present sample indicates that in future research, larger samples are needed to differentiate between categories of professional care and to explore their interrelatedness.

Third, we had only a very rough indicator of social support at our disposal. Respondents were simply asked whether they could turn to friends and to family for first health support. However, social support is important to divorced or separated people for coping with their emotional and psychological problems, even prior to an escalation of those problems.

Fourth, we faced some limitations concerning time. This analysis is based on cross-sectional data, which may hinder a causal interpretation of the results. This is problematic when considering the association between (mental) health status and professional care-seeking behavior. Physical and mental health status was measured at the time of the interview, while the use of mental health care in the 12 months preceding the interview was measured retrospectively. Nevertheless, the former measurement is considered a predictor of the latter. Longitudinal data might help circumvent this problem, but cross-national longitudinal datasets containing information on marital status, mental health status, and mental health professional care seeking are currently nonexistent. In addition, we cannot rule out that possible selection effects may account for the association between marital status and professional care seeking, as previous research has indicated that at least a part of the relation between divorce and mental health is due to selection effects [9,14,77,78]. However, as our results on professional care use for mental health problems are in line with the only longitudinal study, to our knowledge, on this topic [9], we believe that a substantial part of the differences in mental health care use is a consequence of the divorce or separation. In addition, because our study measured professional care use during the 12 months preceding the interview, while the health status indicators referred to only the last 4 weeks, the present results potentially underestimate the importance of the latter. It is unlikely, however, that the lack of comparable time frames explains the marital status differences in professional care seeking. For instance, the present indicator of mental and somatic health status is able to statistically explain the professional care use among the widowed; therefore we could expect it to be a suitable need indicator for the divorced as well.

Finally, due to data limitations we could only consider current marital status. This prevents us from distinguishing between past and current marital status. As a result, we are unable to separate, for instance, married persons from married persons who were previously divorced.

Despite these limitations, our results offer at least three important insights. First, in accordance with previous research [9,10,15,21], we found that the divorced or separated, compared to the married or cohabiting, do have a higher mental health care use, and that differences between the two groups can be only partly explained by differences in physical and mental health. Our findings are based on a representative sample of the population of 29 European countries. They confirm that the more frequent professional care use among the divorced or separated is present in most of the participating countries, albeit to varying degrees. Mental health status has a strong impact on mental health care use and explains professional care seeking among the widowed, and, to a substantial degree, among women. The finding that need factors can explain health service use among the widowed but not among the divorced corroborates previous research by Prigerson et al. [9]. These differential effects confirm that a substantial part of the mental health care use for emotional or psychological problems among the divorced or separated relates to factors--what Andersen calls "need factors"--other than somatic and mental health status. However, our predisposing characteristics and enabling resources, like social conditions related to level of education, work status, age, and the perceived availability of support from friends and family, cannot account for this marital status effect either. Consequently, the finding that the divorced or separated use mental health services more often than the married or cohabiting may indicate the power of relationship loss, bereavement, strife, or social impairment as motivators for seeking treatment (see also [21,22]).

Additionally, we found that cross-national differences in the association between marital dissolution and professional care use are considerable and are linked to the average divorce rate in the participating countries. The higher the divorce rate, the smaller the gap in professional care use between the divorced or separated and the married or cohabiting. To the best of our knowledge, this study is the first to document this cross-level interaction effect. Even more fascinating is the finding that the diminished marital status gap in countries with high average divorce rates results from both an increased professional care use for emotional or psychological problems among married or unmarried couples and a decrease in this help-seeking behavior among the divorced. Gelissen [63] found that the degree of tolerance towards divorce correlates with the national rate of divorce. Therefore, the normalization of divorce [64] may account for less professional care seeking after marital dissolution in countries with high divorce rates. In these countries, divorce is probably less stigmatizing, or is seen as less intrinsically problematic, leading to a net decline in professional care seeking. A post hoc interpretation of the increase in professional care seeking among the married or cohabiting may be that in countries with high divorce rates changes in the meaning of marriage and divorce [63,65-67] may lead both to a higher propensity among couples to proactively contact professional care providers for marriage and relationship counseling and to less reliance on professional support in the aftermath of a marriage breakup. In societies with high divorce rates, a divorce culture gives way to a therapeutic culture [67] in which the married also rely on mental health professionals for counseling more frequently. It is, in part, because of these opposing effects of the national divorce rates on the professional care seeking of the divorced or separated versus the married or cohabiting that we do not find overall effects of the national rates of divorce on the mental health professional support-seeking rate. Because our analyses are based on a comparison of current marital status categories, we cannot determine whether this finding results from the impact of remarriage after divorce. It is still possible that the increase of professional care seeking among the married in countries with high divorce rates is confined to those who remarried after divorce. Analyses of cross-national datasets containing detailed information about marital status transitions are needed to solve this problem.

Finally, gender and work status prove to be important determinants for mental health care use, while age and education do not seem to matter. In line with earlier studies, we found that women use more mental health care than men do. In line with the findings of Koopmans and Lamers [26], although contrary to other research [68], we also found that in most countries this higher mental health care use among women can be completely attributed to self-reported poor mental and physical health. Our hypothesis that the gap in mental health care use between the divorced and married would be greater for women than for men could not be confirmed. Nevertheless, a significant amount of between-country variation in the gendered effect of marital status on professional care seeking was present, suggesting that in some countries divorce leads to increased professional care seeking among women, while in other countries a reversed gender difference can be observed. We did not find any effects from education, presence of children in the household, or social support. These results contrast with what we might expect to find [4,19,20,23,26,69]. When considering the lack of an effect for the presence of children, however, it is possible that two opposite forces are at work: A negative "burden" effect of the presence of children in the household [21,22] may be neutralized by a more positive effect that comes from the support children may give to their parents [16-18]. Not having custody of one's children can be a significant stress factor as well and thus a reason to seek professional care. This is especially relevant for divorced fathers, who often experience significantly decreased contact with their children. Within the divorced group, more detailed analyses are needed to refine these diverging effects. Social support, measured as first health support, does not have a strong influence on the consumption of different health care providers. We find a significant but less substantial effect of the availability of friends as first health supporters, suggesting that the availability of support from laypersons actually enhances professional care consumption. Friends help a person gain insight into personal and emotional problems and stimulate a person to seek professional care [30,33].

## Conclusions

To the best of our knowledge, this is the first comprehensive study of a large European sample that focuses on the differences in mental health service use between the divorced or separated and the married or cohabiting and that considers both individual and country characteristics. The present study has shown that the higher health care use for emotional or psychological problems by the divorced and separated is only partly explained by worse mental and physical health. In addition, our research clearly shows that it is important to consider differences at the country level when studying marital status differences in professional care use: In countries with a high national divorce rate, the gap between the married or cohabiting and the divorced or separated regarding use of professional mental health care diminishes dramatically. Married and unmarried couples are more frequent user of mental health professional support, while the consumption of professional care among the divorced or separated is lower than in countries where divorce occurs less frequently.

In sum, we have reason to assume that in countries with high divorce rates, those seeking professional support for mental health problems are more proactive, while in countries where divorce is less common, the divorced seek professional support to cope with the detrimental consequences of marital dissolution.

## Competing interests

The authors declare that they have no competing interests.

## Authors' contributions

PB was responsible for general coordination, participated in the statistical analysis, supervised the drafting of the manuscript, and finalized the manuscript. EC participated in the literature review and drafted the manuscript. SS made critical revisions to the manuscript. LVP participated in the statistical analysis and the preparation of the first draft of the manuscript. All authors read and approved the final manuscript.

## Pre-publication history

The pre-publication history for this paper can be accessed here:

http://www.biomedcentral.com/1471-2458/10/224/prepub
